# Radiation Exposure with Self-Expandable Metallic Stent versus Transanal Decompression Tube for Malignant Colorectal Obstruction: A Post Hoc Propensity Score Matched Analysis

**DOI:** 10.3390/jcm13195924

**Published:** 2024-10-04

**Authors:** Yuzuru Tamaru, Toshio Kuwai, Shiro Hayashi, Koji Nagaike, Takayuki Yakushijin, Satoshi Asai, Masashi Yamamoto, Shinjiro Yamaguchi, Takuya Yamada, Kenkei Hasatani, Hideyuki Ihara, Hidetaka Tsumura, Hisashi Doyama, Iruru Maetani, Toshio Fujisawa, Yukiko Ito, Tadayuki Takagi, Yasuki Hori, Mamoru Takenaka, Makoto Hosono, Tsutomu Nishida

**Affiliations:** 1 Department of Endoscopy, NHO Kure Medical Center and Chugoku Cancer Center, Kure 737-0023, Japan; tamaru.yuzuru.vh@mail.hosp.go.jp; 2 Department of Gastroenterology, NHO Kure Medical Center and Chugoku Cancer Center, Kure 737-0023, Japan; 3 Gastrointestinal Endoscopy and Medicine, Hiroshima University Hospital, Hiroshima 734-8551, Japan; 4 Department of Gastroenterology and Internal Medicine, Hayashi Clinic, Suita 565-0842, Japan; hayashishiro1976@yahoo.co.jp; 5 Department of Gastroenterology, Toyonaka Municipal Hospital, Toyonaka 560-8565, Japan; yamamasa@gh.med.osaka-u.ac.jp (M.Y.); tnishida.gastro@gmail.com (T.N.); 6 Department of Gastroenterology and Hepatology, Suita Municipal Hospital, Suita 565-0842, Japan; nagaike.koji@gmail.com; 7 Department of Gastroenterology and Hepatology, Osaka General Medical Center, Osaka 558-8558, Japan; yakushijin@gh.opho.jp; 8 Department of Gastroenterology, Tane General Hospital, Osaka 550-0025, Japan; bonyaritetsu1226@hotmail.co.jp; 9 Department of Gastroenterology and Hepatology, Kansai Rosai Hospital, Amagasaki 660-8511, Japan; smay-0608@diary.ocn.ne.jp; 10 Department of Gastroenterology and Hepatology, Osaka Rosai Hospital, Sakai 591-8025, Japan; yamtak1973@gmail.com; 11 Department of Gastroenterology, Fukui Prefectural Hospital, Fukui 910-8526, Japan; hasatani9@yahoo.co.jp; 12 Department of Gastroenterology, Tonan Hospital, Sapporo 060-0004, Japan; h-ihara@tonan.gr.jp; 13 Department of Gastroenterological Oncology, Hyogo Cancer Center, Akashi 673-0021, Japan; h.tsumura@hp.pref.hyogo.jp; 14 Department of Gastroenterology, Ishikawa Prefectural Central Hospital, Kanazawa 920-8530, Japan; doyama.134@gmail.com; 15 Department of Gastroenterology, Sin-Kuki General Hospital, Kuki 346-8530, Japan; mtnir50637@med.toho-u.ac.jp; 16 Department of Gastroenterology, Graduate School of Medicine, Juntendo University, Tokyo 113-8421, Japan; t-fujisawa@juntendo.ac.jp; 17 Department of Gastroenterology, Japanese Red Cross Medical Center, Tokyo 150-8935, Japan; yukikomd1224@gmail.com; 18 Department of Gastroenterology, Fukushima Medical University School of Medicine, Fukushima 960-1295, Japan; daccho@fmu.ac.jp; 19 Department of Gastroenterology and Metabolism, Nagoya City University Graduate School of Medical Sciences, Nagoya 464-8601, Japan; yhori@med.nagoya-cu.ac.jp; 20 Department of Gastroenterology and Hepatology, Kindai University, Faculty of Medicine, Osaka-Sayama 589-8511, Japan; mamoxyo45@gmail.com; 21 Department of Radiology, Faculty of Medicine, Kindai University, Osaka-Sayama 589-8511, Japan; hosono@med.kindai.ac.jp

**Keywords:** radiation exposure, self-expandable metallic stent, transanal decompression tube, rectum, malignant colorectal obstruction

## Abstract

**Background:** Although several reports have compared the outcomes of self-expandable metallic stent (SEMSs) and transanal decompression tube (TDT) placement for malignant colorectal obstruction (MCO), few studies have compared the radiation exposure (RE) associated with these two procedures. Consequently, we aimed to compare the RE of SEMS and TDT placements for MCO using propensity score matching (PSM) in a multi-center, prospective observational study. **Methods:** This study investigated the clinical data of 236 patients who underwent SEMS or TDT placement. The air kerma at the patient entrance reference point (K_a,r_: mGy) and air kerma–area product (P_KA_; Gycm^2^) were measured and compared between SEMS and TDT groups after PSM. **Results:** After PSM, 61 patients were identified in each group. The median K_a,r_ in the SEMS group was significantly greater than that in the TDT group (77.4 vs. 55.6 mGy; *p* = 0.025) across the entire cohort. With respect to subgroup analyses by location, in the rectum, the median K_a, r_ and P_KA_ were significantly greater in the SEMS group than in the TDT group (172.9 vs. 34.6 mGy; *p* = 0.001; and 46.0 vs. 18.1 Gycm^2^; *p* = 0.006, respectively). However, in the colon, the RE parameters did not significantly differ between the two groups. **Conclusions:** TDT might be a more suitable option for decompression in patients with malignant rectal obstruction due to its lower RE and technical advantages. Conversely, SEMS placement is recommended as the first decompression method to treat malignant colonic obstruction, in line with the current guidelines.

## 1. Introduction

Malignant colorectal obstructions (MCOs) are complications caused by primary colorectal cancer or extracolonic malignancies, such as gastric, pancreaticobiliary, and gynecologic cancers. These obstructions are identified in approximately 10% of advanced primary colorectal cancer patients [1,2,3,4,5]. Patients require emergency decompressive procedures to prevent severe complications such as bacterial translocation, electrolyte and fluid imbalance, colonic necrosis, and perforation, which can lead to severe symptoms such as nausea, vomiting, and abdominal pain [6]. Intestinal decompression using a self-expandable metallic stent (SEMS) or a transanal decompression tube (TDT) serves as an alternative to emergency surgery for MCOs [4,6,7,8,9,10,11], both of which require fluoroscopic guidance [3].

Adherence to the appropriate radiation exposure doses is essential for the radiation safety management of patients. The International Commission on Radiological Protection (ICRP) has established diagnostic reference levels (DRLs) [12], which are globally recognized as the standard for procedures involving ionizing radiation [13,14]. These standards, including those established in Japan, are pivotal in ensuring patient safety. However, although several reports have compared the treatment outcomes of SEMS and TDT placements for MCOs [11,15,16], studies on the radiation exposure associated with these procedures are rare.

Therefore, we conducted a post hoc propensity score matching (PSM) analysis using data from a multi-center prospective observational study (the REX-GI study [17]). This analysis aims to evaluate and compare the radiation exposure of SEMS and TDT placement procedures to provide crucial insights for informing treatment decisions and understanding their clinical implications.

## 2. Materials and Methods

### 2.1. Study Design

This study was a post hoc analysis of a multi-center, prospective observational REX-GI study from May 2019 to December 2020 [17,18,19]. The protocol was approved by the institutional review board of the Kure Medical Center and Chugoku Cancer Center (Approval number: 2019-17) and registered with the UMIN Clinical Trials Registry (UMIN000036525). All authors accessed the study data and reviewed and approved the final manuscript. This study was conducted in accordance with the Helsinki Declaration and its later amendments, and the requirement for informed consent was waived using the opt-out method of each hospital website.

### 2.2. Patients, Outcomes and Definitions

**Figure 1:**
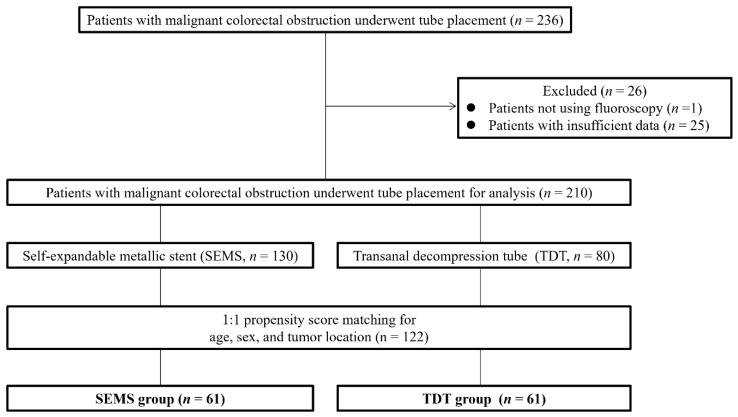
Patient flowchart. SEMS, self-expandable metallic stent; TDT, transanal decompression tube.

In the original REX-GI study, 236 consecutive patients with MCO underwent SEMS or TDT placement between May 2019 and December 2020 (Figure 1). Excluding one patient whose procedure was not guided by fluoroscopy and twenty-five patients whose data were insufficient, the analysis included 210 patients (130 in the SEMS group and 80 in the TDT group). A PSM analysis was conducted to minimize confounding bias. The variables to estimate the propensity score were age, sex, and tumor location. Thereafter, 1:1 nearest neighbor matching was performed using a caliper set at 0.25, which resulted in 61 patients in each group for analysis (Table 1). We assessed the air kerma at the patient entrance reference point (K_a,r_: mGy), air kerma–area product (P_KA_; Gycm^2^), fluoroscopy time (FT; min), and procedure time (PT; min). The primary outcome was to compare K_a,r_, P_KA_, FT, and PT between SEMS and TDT groups, and the secondary outcomes included comparisons based on the colorectum location. K_a,r_ is the intensity when the X-ray beam from the fluoroscope collides with the air, and P_KA_ is the product of K_a,r_ and the X-ray beam area perpendicular to the beam axis [12]. The colon was defined as the region from the cecum to the sigmoid colon (including the sigmoid colon). The right-sided colon was defined as the region from the cecum to the transverse colon, and the left-sided colon was defined as the region from the descending colon to the sigmoid colon. The rectum was defined as the region from the rectum to the dentate line.

### 2.3. Statistical Analysis

Categorical and nominal variables are expressed as numbers and percentages, whereas continuous variables are expressed as medians and interquartile ranges (IQRs). A two-sided *p* value less than 0.05 was considered to indicate statistical significance. All statistical analyses were performed using EZR version 1.52 (Saitama Medical Center, Jichi Medical University, Saitama, Japan), which is a graphical user interface for R version 4.02 (the R Foundation for Statistical Computing, Vienna, Austria) [20].

## 3. Results

### 3.1. Clinical Characteristics of the Patients and Lesions

Table 1 shows the clinical characteristics of the studied patients and lesions. Among the 210 patients, the median age was 72.5 years, 48.1% (101/210) were male, and 79.5% (167/210) had tumors localized in the colon. Prior to PSM, the SEMS group had a greater median age (74.0 years) and greater incidence of colon cancer (90.0% [117/130]) than the TDT group (71.0 years and 62.5% [50/80], respectively; *p* = 0.002 and *p* < 0.001). The sex distribution was similar between the two groups (*p* = 0.570). After PSM, there were no significant differences in clinical characteristics between the two groups.

### 3.2. Radiation Exposure

**Figure 2:**
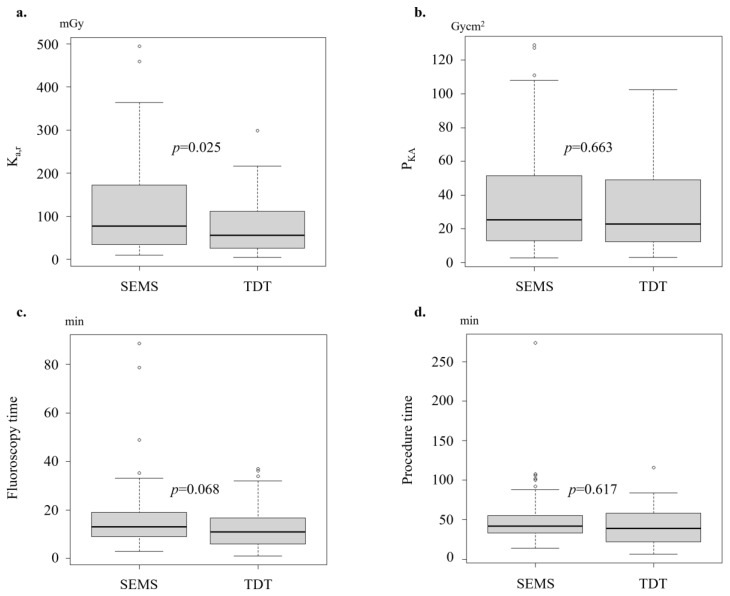
Comparison of the radiation exposure (K_a,r_ (**a**), P_KA_ (**b**)), fluoroscopy time (**c**), and total procedure time (**d**) (after propensity score matching; entire cohort).

Post-PSM, the median K_a,r_ was significantly greater in the SEMS group (77.4 mGy) than in the TDT group (55.6 mGy; *p* = 0.025) (Table 2, Figure 2). The median FT tended to be longer in the SEMS group than in the TDT group (13.0 vs. 10.9 min; *p* = 0.068). However, there were no significant differences in the median P_KA_ (25.3 vs. 23.0 Gycm^2^; *p* = 0.663) or PT (24.0 vs. 26.0 min; *p* = 0.617) measurements. Before PSM, there were no significant differences in procedural outcomes between the groups.

### 3.3. Subgroup Analyses of Radiation Exposure by Location

**Figure 3:**
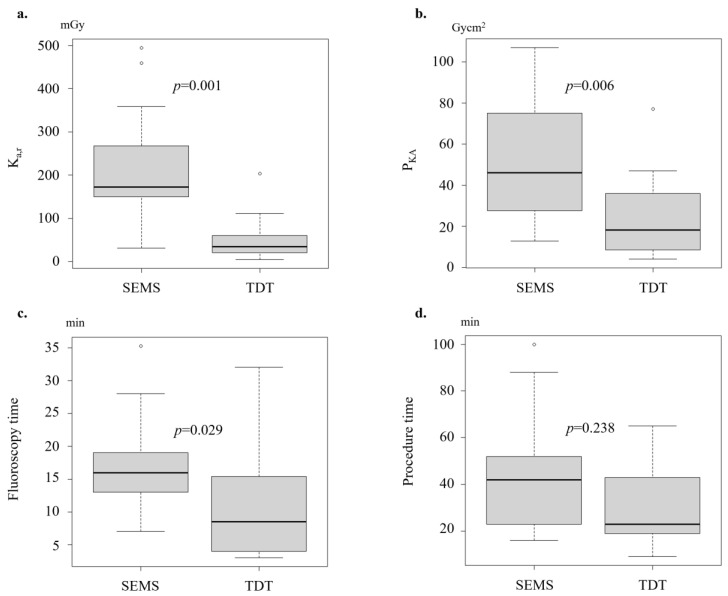
Comparison of the radiation exposure (K_a,r_ (**a**), P_KA_ (**b**)), fluoroscopy time (**c**), and total procedure time (**d**) (after propensity score matching; rectum).

According to our subgroup analyses on the rectum location, the patients in the SEMS group had significantly greater median K_a,r_ and P_KA_ values (172.9 vs. 34.6 mGy; *p* = 0.001; and 46.0 vs. 18.1 Gycm^2^; *p* = 0.006, respectively) than those in the TDT group after PSM (Table 3, Figure 3). Additionally, the median FT was significantly longer in the SEMS group than in the TDT group (16.0 vs. 8.5 min; *p* = 0.029). In contrast, in the colon cancer subgroup, there were no significant differences in radiation exposure parameters between the two groups.

## 4. Discussion

In this post hoc PSM analysis of the multi-center prospective observational REX study, we observed that SEMS placement resulted in significantly greater K_a,r_ values and longer fluoroscopy times than the TDT placement. Notably, these differences were more pronounced for rectal lesions than for colonic lesions.

Specifically, for rectal lesions, K_a,r_, P_KA_ and FT were significantly greater in the SEMS group than in the TDT group (K_a,r_: 172.9 vs. 34.6 mGy; *p* = 0.001; P_KA_: 46.0 vs. 18.1 Gycm^2^; *p* = 0.006; FT: 16.0 vs. 8.5 min; *p* = 0.029). This disparity is likely attributed to the technical complexities in the SEMS placement for rectal lesions, whereas TDT placement is relatively easy. The TDT insertion technique involves using a colonoscope to identify the obstruction site, injecting a water-soluble contrast medium to delineate the stricture, and advancing a guide wire through the tumor beyond the point of obstruction under fluoroscopic and endoscopic guidance. The colonoscope was withdrawn, and the TDT was placed over the guide wire after the colonoscope had been withdrawn; the balloon of the TDT was inflated with distilled water to prevent migration [10]. For rectal lesions, a shorter distance from the anus to the lesion facilitates the TDT placement, which reduces both procedure time and fluoroscopy time. In contrast, rectal stenting is less successful in patients with tumors near the anal verge and is often avoided due to the presumed association with complications such as pain, tenesmus, incontinence, and stent migration [21,22]. In addition, for bridge-to-surgery stenting, placing a SEMS in the rectum may interfere with the primary anastomosis and cause stoma creation if the position of the SEMS placement slightly shifts toward the anal side. Therefore, the position of the distal edge of the stent must be carefully considered, which is technically challenging and may result in a significantly longer FT (16.0 vs. 8.5 min; *p* = 0.029) and a greater radiation dose (K_a,r_: 172.9 vs. 34.6 mGy; *p* = 0.001; P_KA_: 46.0 vs. 18.1 Gycm^2^; *p* = 0.006) in the present study. Comparatively, the K_a,r_ value for patients treated with barium enema, as defined in Japan DRLs 2020, was 130 mGy [13]. Radiation exposure should be considered for both patients and operators during fluoroscopy. Considering the undetermined efficacy of the SEMS placement for rectal lesions and the findings of this study, TDT placement may be a valid option to treat rectal lesions, although TDT placement is not recommended in the European Society of Gastrointestinal Endoscopy (ESGE) guidelines [3].

Although there was a significant difference in radiation dose (K_a,r_) and a tendency toward longer FT in the entire PSM cohort (colon and rectum), there were almost no differences regarding radiation dose (K_a,r_ and P_KA_), FT, or PT between the two groups in the colon only (K_a,r_: 64.0 vs. 57.4 mGy; *p* = 0.524; P_KA_: 21.6 vs. 26.7 Gycm^2^; *p* = 0.303; FT; 12.0 vs. 11.0 min; *p* = 0.317; PT: 42.5 vs. 40.0 min; *p* = 0.250). A recent study on the short-term outcomes (success rates and adverse events) of SEMS and TDT placements reported that the clinical success rate was significantly lower in the TDT group than in the SEMS group (85.9% vs. 97.3%; *p* = 0.004), and significantly more patients required emergency surgery in the TDT group than in the SEMS group (12.9% vs. 2.7%; *p* = 0.009) [16]. Furthermore, a recent meta-analysis reported lower technical/clinical success rates, fewer primary tumor resections/anastomoses, and more stomas for the TDT placement than for the SEMS placement [11]. Therefore, considering the results regarding radiation exposure in this study, the SEMS placement is considered the first decompression method to treat a malignant colonic obstruction, as indicated in the ESGE guidelines [3].

This study has several limitations. First, this study was not a randomized controlled trial but a post hoc analysis based on an observational cohort with a limited number of clinical records (not including the short- and long-term outcomes of each procedure). Second, this study included patients from the same ethnic population who shared similar geographic and healthcare settings. Third, this study might have been affected by selection bias because the patients and procedures, including the selection of procedures, were not randomized. Therefore, the background information of the patients was adjusted by propensity score matching. Fourth, there might be insufficient power due to the small number of enrolled patients. Although there have been some reports on radiation exposure during endoscopic procedures, there are only a few reports of radiation exposure during SEMS placements [23,24], and there are currently no reports of radiation exposure during TDT placements. Furthermore, no studies compared the radiation exposure between SEMS and TDT placements, which makes this work the first such study.

In conclusion, for endoscopic decompression to correct MCOs, radiation exposure during SEMS placements was greater than that during TDT placements, especially in the rectum. Additionally, the effectiveness of the SEMS placement for rectal lesions has not been fully elucidated, and this procedure is considered technically challenging. Therefore, TDT might be a preferable decompression option for malignant rectal obstruction due to the associated lower radiation exposure and beneficial technical aspects. On the other hand, for malignant colonic obstruction, SEMS is considered the first decompression method, as indicated in the current guidelines.

## Figures and Tables

**Table 1:** Clinical characteristics of the studied patients and lesions.

Variables	All Cohort (*n* = 210)			Propensity-Matched Cohort (*n* = 122)		
	SEMS Group (*n* = 130)	TDT Group (*n* = 80)	*p* Value	SEMS Group (*n* = 61)	TDT Group (*n* = 61)	*p* Value
Age, median (IQR), years	74.0 (67.0–84.0)	71.0 (54.8–77.0)	0.002	70.0 (64.0–81.0)	73.0 (57.0–81.0)	0.665
Sex, male, *n* (%)	65 (50.0)	36 (45.0)	0.570	29 (47.5)	31 (50.8)	0.856
Location, colon, *n* (%)	117 (90.0)	50 (62.5)	˂0.001	48 (78.7)	48 (78.7)	1.000
Right side	38 (29.2)	8 (10.0)		6 (6.8)	8 (13.1)	
Left side	79 (60.8)	42 (52.5)		42 (68.9)	40 (65.6)	

SEMS: self-expandable metallic stent; TDT: transanal decompression tube.

**Table 2:** Comparison of procedure details between patients in the SEMS and TDT groups.

Variables	Before Propensity Score Matching				After Propensity Score Matching			
	Total (*n* = 210)	SEMS Group (*n* = 130)	TDT Group (*n* = 80)	*p* Value	Total (*n* = 122)	SEMS Group (*n* = 61)	TDT Group (*n* = 61)	*p* Value
K_a,r_, median (IQR), mGy	60.6 (32.9–124.6)	62.0 (33.8–124.9)	57.4 (26.0–123.0)	0.245	63.5 (30.2–148.2)	77.4 (34.1–172.5)	55.6 (26.0–111.2)	0.025
P_KA_, median (IQR), Gycm^2^	23.0 (13.1–49.8)	22.7 (13.5–49.8)	25.2 (12.8–49.2)	0.814	24.4 (12.3–50.0)	25.3 (12.9–51.6)	23.0 (12.3–48.9)	0.663
Fluoroscopy time, median (IQR), min	11.9 (8.0–17.0)	12.6 (9.0–18.0)	11.0 (6.2–16.6)	0.119	11.8 (8.0–18.0)	13.0 (9.0–19.0)	10.9 (6.0–16.7)	0.068
Procedure time, median (IQR), min	40.0 (26.0–56.8)	40.0 (31.3–55.8)	38.5 (23.0–57.3)	0.103	40.0 (26.0–57.8)	24.0 (20.0–36.0)	26.0 (14.0–39.0)	0.617

*IQR*: interquartile range; *K_a,r_*: air kerma at the patient entrance reference point; *P_KA_*: air kerma-area product; *SEMS*: self-expandable metallic stent; *TDT*: transanal decompression tube.

**Table 3:** Comparison of procedure details between patients in the SEMS and TDT groups (based on the location in the colorectum).

Variables	SEMS Group	TDT Group	*p* Value
Colon, *n*	48	48	
K_a,r_, median (IQR), mGy	64.0 (31.8–125.6)	57.4 (26.0–124.5)	0.524
P_KA_, median (IQR), Gycm^2^	21.6 (11.0–49.3)	26.7 (14.0–54.9)	0.303
Fluoroscopy time, median (IQR), min	12.0 (9.0–18.3)	11.0 (6.5–18.5)	0.317
Procedure time, median (IQR), min	42.5 (33.0–56.5)	40.0 (25.0–58.3)	0.250
Rectum, *n*	13	13	
K_a,r_, median (IQR), mGy	172.9 (149.8–268.0)	34.6 (21.2–60.3)	0.001
P_KA_, median (IQR), Gycm^2^	46.0 (27.6–75.0)	18.1 (8.5–35.8)	0.006
Fluoroscopy time, median (IQR), min	16.0 (13.0–19.0)	8.5 (4.0–15.4)	0.029
Procedure time, median (IQR), min	42.0 (23.0–52.0)	23.0 (19.0–43.0)	0.238

*IQR*: interquartile range; *K_a,r_*: air kerma at the patient entrance reference point; *P_KA_*: air kerma-area product; *SEMS*: self-expandable metallic stent; *TDT*: transanal decompression tube.

## Data Availability

The data that support the findings of this study are available upon request from the corresponding author (Toshio Kuwai).
